# Self-modulating therapeutic platform using engineered miRNA-responsive oligonucleotides

**DOI:** 10.1186/s40580-025-00499-w

**Published:** 2025-06-30

**Authors:** Doyeong Ku, Hansol Kim, JinA Lim, Jayeon Song, Junhyeok Yoon, Liu Jun, Su-Ji Min, Ryeongeun Cho, Namseok Lee, Kyunghoon Hur, Jong-Eun Park, Luke P. Lee, Junshik Hong, Yoosik Kim, Hyun Gyu Park

**Affiliations:** 1 Department of Chemical and Biomolecular Engineering (BK 21+ Program), Korea Advanced Institute of Science and Technology (KAIST), Daejeon, 34141 Republic of Korea; 2 Department of Internal Medicine, Seoul National University College of Medicine, Seoul, 03080 Republic of Korea; 3 Cancer Research Institute, Seoul National University College of Medicine, Seoul, 03080 Republic of Korea; 4 Graduate School of Medical Science and Engineering, KAIST, Daejeon, 34141 Republic of Korea; 5 Department of Medicine, Harvard Medical School, Brigham Women’s Hospital, Boston, MA USA; 6 Department of Bioengineering, Department of Electrical Engineering and Computer Science, University of California at Berkeley, Berkeley, CA USA; 7 Institute of Quantum Biophysics, Department of Biophysics, Sungkyunkwan University, Suwon, Republic of Korea; 8 Biomedical Research Institute, Seoul National University College of Medicine, Seoul, 03080 Republic of Korea; 9 KAIST Institute for BioCentury, KAIST, Daejeon, 34141 Republic of Korea; 10 KAIST Institute for Health Science and Technology (KIHST), KAIST, Daejeon, 34141 Republic of Korea

**Keywords:** miRNAs, Apoptosis induction, Gene regulation, BCL-xL gene, Oligonucleotide therapy

## Abstract

Due to a pivotal role in the post-transcriptional regulation of genes implicated in numerous diseases, miRNAs serve as promising disease biomarkers and therapeutic targets. We introduce a new oligonucleotide probe termed miRNA-trigger, which selectively downregulates newly assigned target mRNAs by hijacking specific miRNAs. By engineering the miRNA-trigger to suppress the anti-apoptotic *BCL-xL* gene, we induce apoptosis selectively in breast cancer cells overexpressing specific miRNAs and further validate its therapeutic efficacy in vivo, by significantly reducing the tumor volume of the xenograft mouse upon its tail-vein injection. This approach establishes a new platform for self-modulating oligonucleotide therapy by redirecting disease-associated miRNAs.

**Graphical Abstract:**

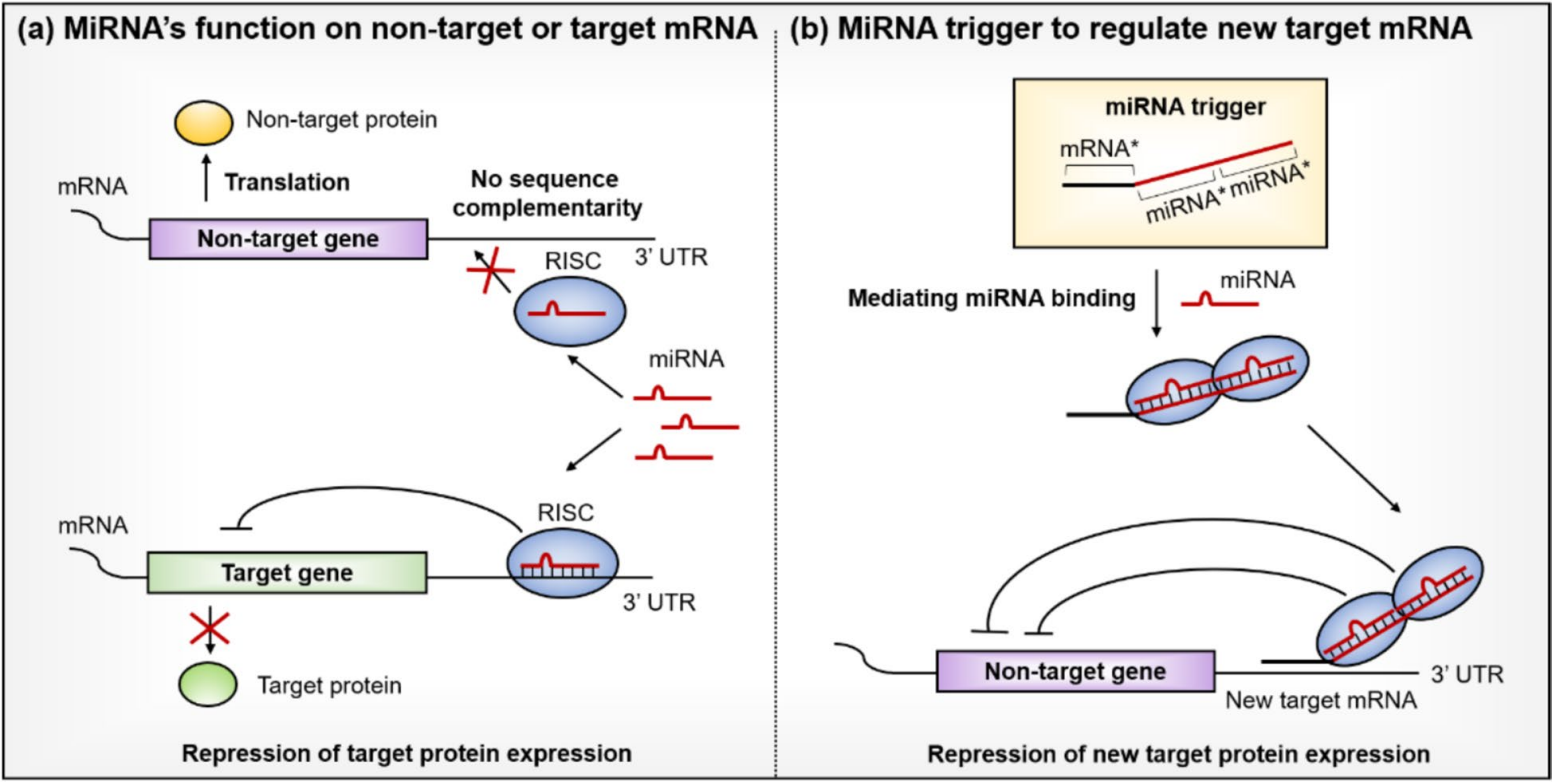

**Supplementary Information:**

The online version contains supplementary material available at 10.1186/s40580-025-00499-w.

## Introduction

During the past decade, emerging evidence has shown that the expression profiles of miRNAs significantly change during disease development and progression, and the dynamic expression levels of miRNAs are implicated in pathogenic conditions of a variety of human diseases, including cancers, heart diseases, neurological diseases, immune function disorders, and age-related diseases [1–7]. Therefore, miRNAs have been regarded as potential biomarkers for early diagnosis of human diseases and prognosis during clinical treatments, and indicators for cellular status [8–10]. Particularly, the profiles of extracellular and circulating miRNAs from readily available fluids such as blood, saliva, and urine have been shown to reflect the status of various human diseases, which has made miRNAs promising non-invasive biomarkers to realize the full potential of liquid biopsy. Consequently, extensive research has been focused on developing the accurate means to quantitate the miRNA expressions [11–14] to make the most of miRNA biomarkers.

The post-transcriptional regulatory functions of miRNAs in human diseases have also made them quite attractive as a novel class of therapeutic targets. They have been developed as agomirs to mimic endogenous miRNAs or antagomirs to inhibit their function [15, 16]. These approaches have shown promise in miRNA-based gene therapy, but they do come with several limitations, particularly concerning potential side effects on normal cells. For instance, agomirs could bind unintended mRNA targets, causing off-target gene suppression that disrupts normal cell functions and leads to side effects in healthy cells [17, 18]. Antagomirs could also cause derepression of genes that miRNAs normally regulate in normal cells, consequently leading to unintended side effects such as promoting unwanted cell proliferation or inhibiting normal apoptosis in healthy tissues [19]. Despite these challenges, miRNAs regulating disease-causing genes in a sequence-specific manner remain highly promising therapeutic targets for personalized treatment. However, improving cell-specific delivery and enhancing target specificity are crucial to minimizing side effects.

Beyond the separate research approaches of using miRNAs as disease biomarkers or gene therapy targets, we have developed a novel RNA therapeutic platform, termed miRNA-trigger, that bridges the monitoring of disease-specific miRNAs with the therapeutic regulation of newly assigned target genes for disease treatment. Upon binding to a specific miRNA, the miRNA-trigger was designed to hijack the miRNA/RISC complex and redirect it to a new target mRNA that does not share sequence complementarity with the miRNA. This technique allows any predetermined genes to be downregulated in order to treat diseases. Importantly, unlike the conventional miRNA-based strategies, such as agomirs and antagomirs, or alternative small RNAs, our system based on the miRNA-trigger induces gene silencing only in cells where a specific disease-associated miRNA is overexpressed, ultimately enabling cell-specific gene therapy [20–22]. By applying this unique design principle to repress the anti-apoptotic B-cell lymphoma-extra-large (*BCL-xL*) gene, we successfully induced apoptosis in target breast cancer cells in a miRNA-dependent manner, both in vitro and in vivo.

Collectively, this work presents the development, engineering, and application of a novel therapeutic platform, termed miRNA-trigger, which enables cell-specific gene regulation, tuned to the expression profiles of disease-specific miRNA.

## Results and discussion

### Design principle of miRNA-trigger for target gene repression in a miRNA-dependent manner

During post-transcriptional regulation, miRNAs just determine their target mRNAs through seed sequence complementarity, while the actual gene silencing is carried out by the associated RISC complex [23]. A previous study showed that anchoring Argonaut (AGO), a miRNA-binding protein and key RISC component, to the 3′UTR of a reporter gene repressed gene expression in a miRNA-independent manner [23]. Inspired by this mechanism, we envisioned that introducing an oligonucleotide that bridges between a specific miRNA and a predetermined mRNA could similarly recruit the RISC complex to a new target, leading to gene silencing.

**Fig. 1:**
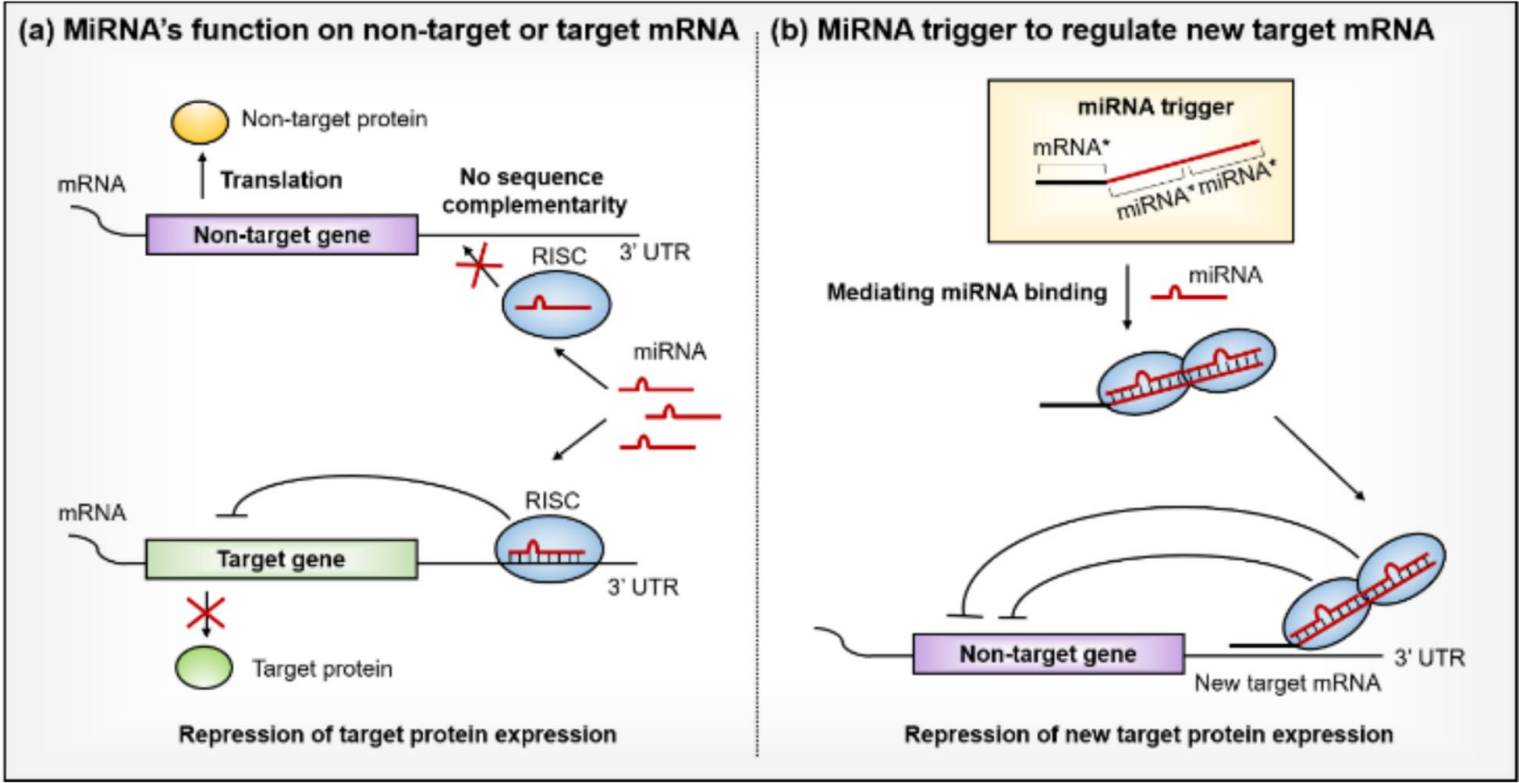
Design principle and proposed mechanism of the miRNA-trigger. MiRNAs guide the RISC complex to complementary sequences in the 3′ UTR of mRNAs to suppress gene expression. Our miRNA-trigger exploits this mechanism by recruiting RISC via tandem miRNA-binding sites and redirecting it to a target mRNA via anti-mRNA sequences

Building on this concept, we developed a miRNA-trigger that utilizes miR-141 or miR-200c to downregulate the *PKR* gene transcript, selected based on our previous findings that *PKR* knockdown has minimal effects on cell proliferation and is easily regulated via RNAi [24]. The miRNA-trigger, approximately 60 nucleotides long, contains two tandem miRNA (miR-141 or miR-200c) binding sites, followed by 20 nucleotide long anti-mRNA sequences designed to bind the 3′UTR of the target (*PKR*) mRNA (Fig. 1). During miRNA biogenesis, each precursor miRNA generates a duplex RNA, which further undergoes strand selection. We identified the dominant strand based on read abundance from the miRbase database and designed 21–23 nucleotides of reverse complementary sequences accordingly for binding. The tandem miRNA complementary binding sites recruit endogenous miRNAs along with their associated RISC complexes, while the adjacent anti-mRNA region hybridizes with the 3′UTR of the target mRNA. This design effectively brings the miRNA-loaded RISC complex to the new target mRNA and promotes its silencing. Preliminary experiments revealed that the tandem miRNA binding sites enhance target mRNA repression by recruiting two miRNAs complexed with RISC to a single miRNA-trigger simultaneously.

### Engineering and validation of miRNA-trigger for cell-specific repression of the *PKR* Gene

**Fig. 2:**
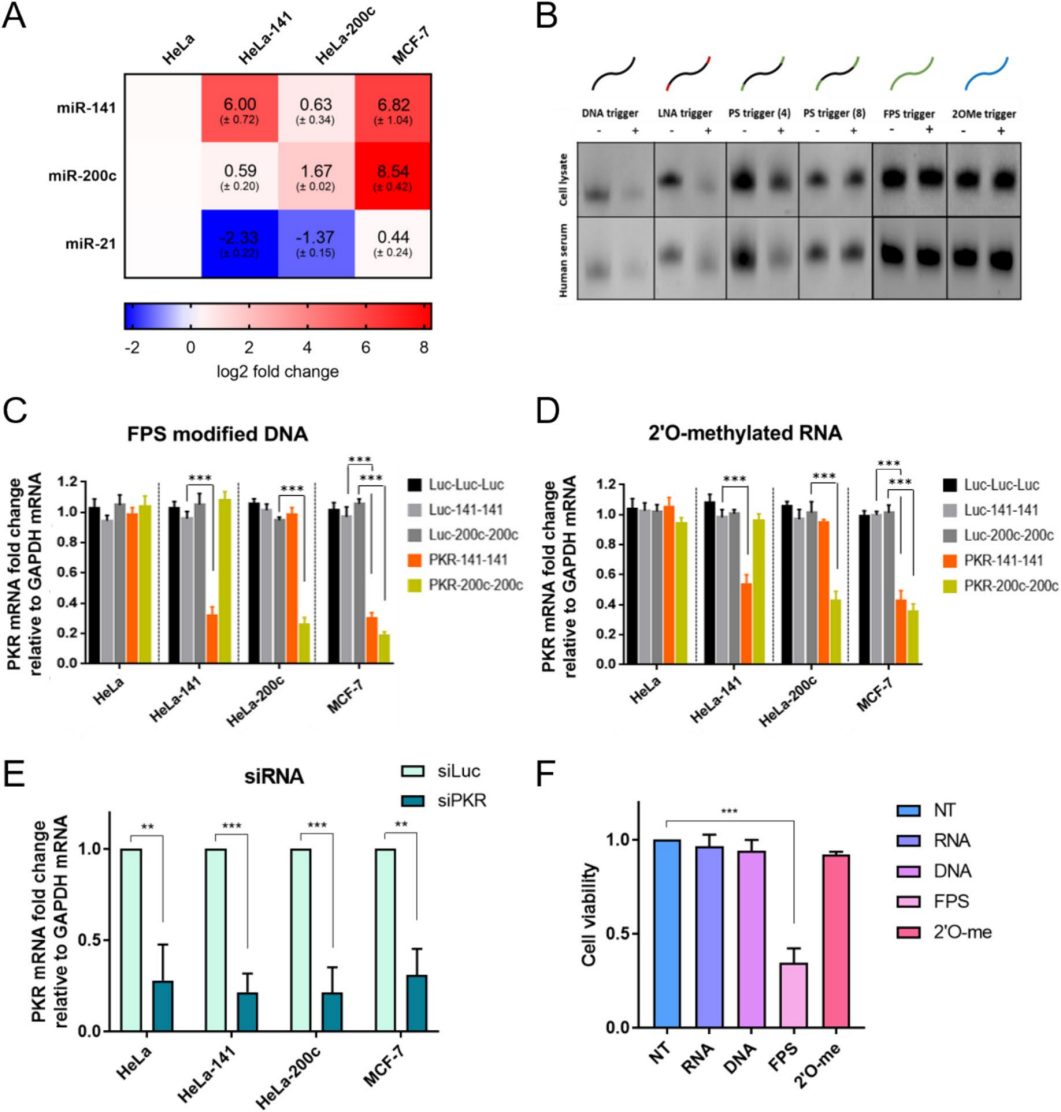
Development of the miRNA-trigger to downregulate *PKR* gene expression. **A** The stem-loop RT-qPCR to profile the miRNA expressions in the four cancer cell lines used in this study. **B** Effects of various chemical modifications on the stability of miRNA-triggers in cell lysates and human serum. **C**–**E** *GAPDH*-normalized *PKR* mRNA expression in HeLa, HeLa-141, HeLa-200c, and MCF-7 cells after transfection with phosphorothioate (PS) modified DNA miRNA-trigger (**C**), 2′O-methylated RNA miRNA-trigger (**D**), and siRNA (**E**). Statistical significance was analyzed using one-tailed Student’s t-tests; * p ≤ 0.05, ** p ≤ 0.01, and *** p ≤ 0.001. **F** Cytotoxicity of chemically modified miRNA-triggers in HeLa cells. Statistical significance was analyzed using a one-way ANOVA with Dunnett’s post hoc test. * adjusted p ≤ 0.05, ** adjusted p ≤ 0.01, and *** adjusted p ≤ 0.001. Unless indicated, the means of three biological replicates were shown, and error bars denote s.e.m

In this study, we utilized HeLa cells with ectopic expression of miR-141 and miR-200c under a constitutive promoter, referred to as HeLa-141 and HeLa-200c, respectively. Additionally, we used MCF-7 cells, which naturally overexpress both miR-141 and miR-200c. The ectopic expression of miR-141 and miR-200c in HeLa-141 and HeLa-200c, as well as the overexpression of both miRNAs in MCF-7 cells, was confirmed using stem-loop RT-qPCR (Fig. 2A).

We developed and transfected *PKR*-targeting miRNA-triggers (PKR-141-141 and PKR-200c-200c) into cells overexpressing miR-141 and/or miR-200c, including HeLa-141, HeLa-200c, and MCF-7. After 48 h, total RNA was extracted, and *PKR* mRNA expression levels were measured. As a control, we designed triggers that bind to miR-141 or miR-200c but target *Luciferase* mRNA (Luc-141-141 or Luc-200c-200c). Additionally, we used a control probe (Luc-Luc-Luc) containing three tandem 20-mer Luc mRNA complementary sequences, designed to interact with neither miRNAs nor mRNAs in the cells, serving as a transfection control. The results showed that the two miRNA-triggers only marginally repressed *PKR* mRNA expression, similar to the three negative control triggers (Figure S1).

We hypothesized that the unexpectedly low gene repression might be due to the instability of the miRNA-trigger. Indeed, when the unmodified 60-mer DNA miRNA-trigger was incubated in human serum and cell lysate for 24 h, about half of it was degraded. To address this stability issue, we incorporated modified nucleotides into the miRNA-triggers and evaluated their effects. Our experiments showed that phosphorothioate (PS) DNA and 2′O-methyl (2′O-Me) RNA modifications significantly improved the stability of the miRNA-trigger. Additionally, we found that modifying all the nucleotides, rather than only a portion, was necessary to make the miRNA-trigger fully resistant to degradation in both cell lysate and human serum (Fig. 2B).

Based on these findings, we synthesized PKR-141-141 and PKR-200c-200c miRNA-triggers using either fully PS-modified DNAs or fully 2′O-Me modified RNAs. We then assessed their effects on *PKR* mRNA expression in HeLa, HeLa-141, HeLa-200c, and MCF-7 cells. As shown in Fig. 2C and 2D, the modified PKR-141-141 triggers effectively downregulated *PKR* gene expression in HeLa-141 and MCF-7 cells but had no effect in wild-type HeLa or HeLa-200c. Similarly, PKR-200c-200c repressed *PKR* mRNA expression in HeLa-200c and MCF-7 cells but not in wild-type HeLa or HeLa-141. Notably, the degree of repression by the miRNA-triggers was similar between HeLa-141 or HeLa-200c and MCF-7, indicating that the miRNA-triggers do not differentiate between exogenous and endogenous miRNAs. Importantly, miR-141 and miR-200c belong to the same miRNA family and share highly similar sequences, differing by only one nucleotide in their seed sequences, with 18 out of 22 nucleotides being identical. Despite this, the PKR-141-141 and PKR-200c-200c triggers precisely discriminated between cells overexpressing the intended miRNA, demonstrating the excellent specificity of this technology.

For comparison, we repeated the experiments using a siRNA targeting the same region of *PKR* mRNA. The siRNA transfection led to significant gene repression in all three cell lines, regardless of the miRNAs expressed (Fig. 2E). This highlights the advantage of the miRNA-trigger, which selectively represses target mRNA in a miRNA-dependent manner, whereas the siRNA indiscriminately suppresses the target mRNA in all cells.

We also evaluated the cytotoxicity of PS and 2′O-Me modifications on HeLa cells and found that the 2′O-Me modification did not induce any significant cytotoxic effects, while the FPS modification significantly reduced cell viability (Fig. 2F). This result is consistent with previous studies that reported cytotoxic effects of the FPS modification, potentially due to non-specific protein binding and cellular stress response associated with the PS backbone chemistry [25, 26]. Therefore, 2′O-Me modified RNA miRNA-triggers were employed for the rest of this study.

### Development and application of miRNA-trigger for cell-specific apoptosis in breast cancer cells

A potential therapeutic application of the developed miRNA-trigger is to hijack oncomirs, inducing apoptosis specifically in cancer cells that overexpress these miRNAs. To achieve this, we applied our technology to target cancer-upregulated miRNAs to downregulate anti-apoptotic *BCL-2* family genes. Numerous studies have developed inhibitors of *BCL-2* family genes or used RNAi approaches to induce apoptosis in cancer cells [27–31]. However, these methods can also promote apoptosis in non-cancerous cells, highlighting the need for more specific techniques that deliver gene-regulating components exclusively to cancer cells. In contrast, our miRNA-trigger selectively targets *BCL-2* family genes only in cancer cells that overexpress the intended miRNAs.

**Fig. 3:**
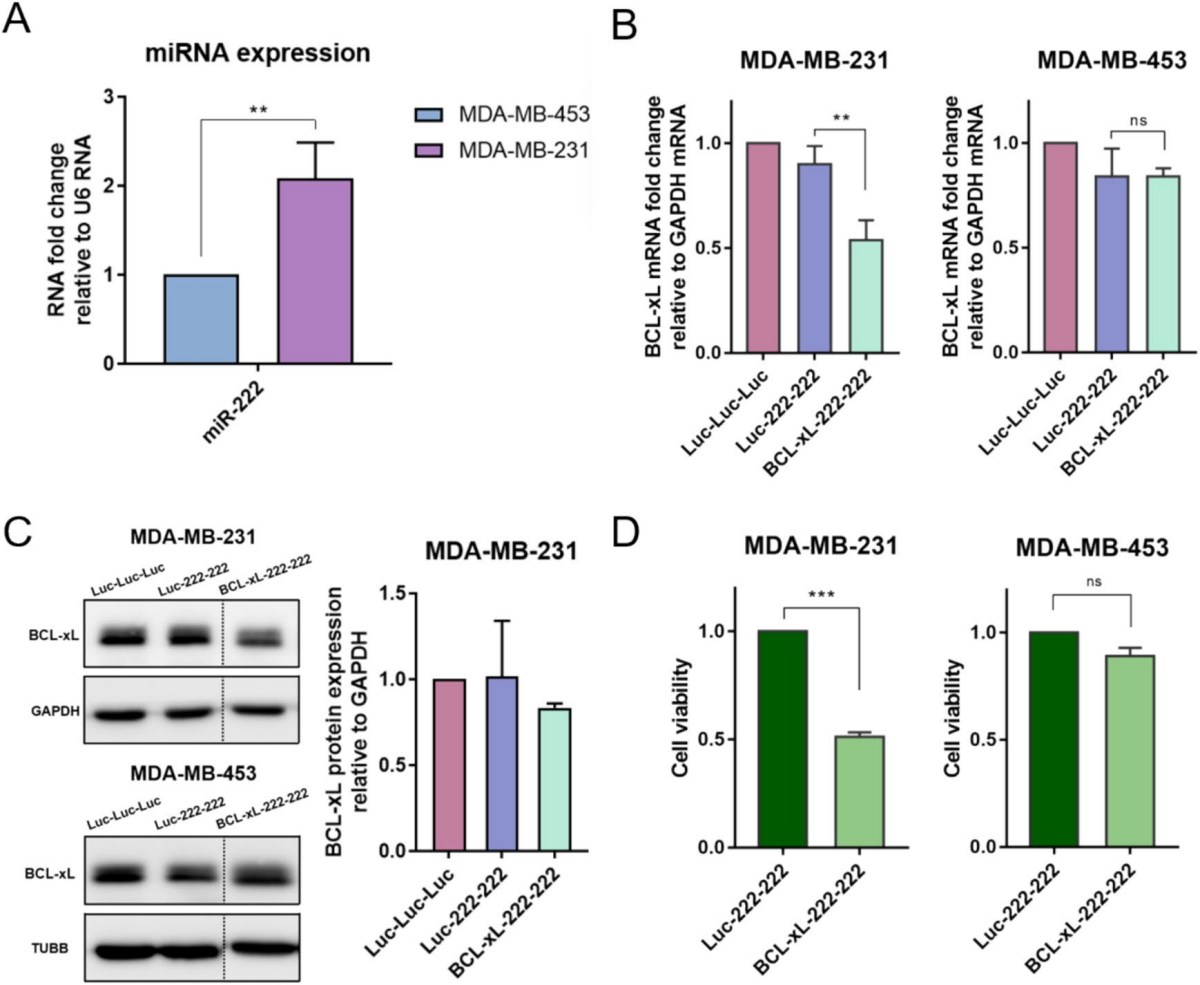
Targeting *BCL-xL* gene to induce cell-specific apoptosis using the miRNA-trigger. **A** *U6* RNA-normalized relative expression of miR-222 in MDA-MB-231 and MDA-MB-453 cells. Statistical significance was analyzed using one-tailed Student’s t-tests; * p ≤ 0.05, ** p ≤ 0.01, and *** p ≤ 0.001. **B** *GAPDH*-normalized *BCL-xL* mRNA expression in MDA-MB-231 and MDA-MB-453 cells after transfection with BCL-xL-222-222 miRNA-trigger. Statistical significance was analyzed using a one-way ANOVA with Dunnett’s post hoc test. * adjusted p ≤ 0.05, ** adjusted p ≤ 0.01, and *** adjusted p ≤ 0.001. **C** Western blot analysis of BCL-xL protein expression in MDA-MB-231 and MDA-MB-453 cells after transfection with BCL-xL-222-222, with GAPDH and TUBB as loading controls for MDA-MB-231 and MDA-MB-453, respectively. Quantification of BCL-xL band intensities is shown on the right. Statistical significance was analyzed using a one-way ANOVA with Dunnett’s post hoc test. **D** Cell viability of MDA-MB-231 and MDA-MB-453 cells after transfection with BCL-xL-222-222. Statistical significance was analyzed using one-tailed Student’s t-tests; * p ≤ 0.05, ** p ≤ 0.01, and *** p ≤ 0.001. Unless indicated, the means of three biological replicates were shown, and error bars denote s.e.m

**Fig. 4:**
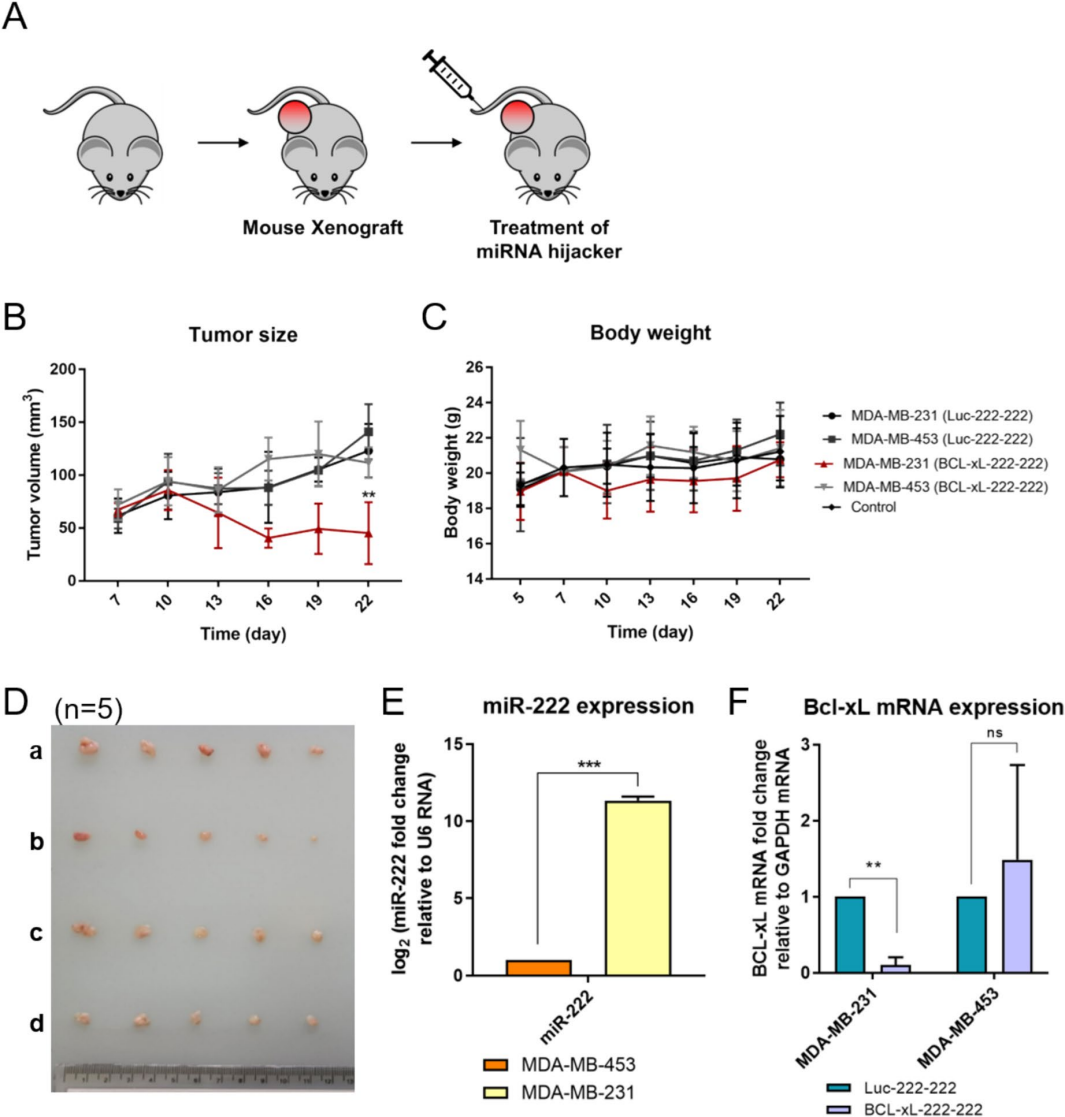
Inducing cell-specific apoptosis using the miRNA-trigger in breast cancer xenograft mouse models. **A** Establishment of breast cancer xenograft mouse model and injection of the miRNA-trigger. **B** Monitoring tumor volume in the breast cancer xenograft mice during treatment with BCL-xL-222-222. **C** Monitoring bodyweight of the mice during treatment with BCL-xL-222-222. **D** Images of tumors excised from MDA-MB-231 or MDA-MB-453 xenograft mice three days after the final injection (day 22) with the BCL-xL-222-222 or negative control Luc-222-222. (**a**: MDA-MB-231 xenograft with Luc-222-222; **b**: MDA-MB-231 xenograft with BCL-xL-222-222; **c**: MDA-MB-453 xenograft with BCL-xL-222-222; **d**: MDA-MB-453 xenograft with Luc-222-222). **E** Expression levels of miR-222 from RNAs extracted from MDA-MB-231 and MDA-MB-453 tumors. **F** *BCL-xL* mRNA expression levels from RNAs extracted from MDA-MB-231 and MDA-MB-453 tumors. Each set of experiments with xenograft mouse models included and analyzed five equivalent breast cancer xenograft mice (**B**–**F**). Error bars denote s.e.m. All of the statistical significance was analyzed using one-tailed Student’s t-tests; *ns *not significant, * p ≤ 0.05, ** p ≤ 0.01, and *** p ≤ 0.001

To demonstrate the cell-specific therapeutic capability, we used two breast cancer cell lines, MDA-MB-231 and MDA-MB-453. Since only MDA-MB-231 cells highly express miR-222 (Fig. 3A) [32], we developed a miR-222 trigger targeting the 3′UTR of *BCL-xL* (BCL-xL-222-222). Transfection of BCL-xL-222-222 significantly downregulated *BCL-xL* mRNA expression in MDA-MB-231 cells, while it had no significant effect in MDA-MB-453 cells (Fig. 3B). Although a reduction in BCL-xL protein level was observed in MDA-MB-231 cells, high variance in the Luc-222-222 control group resulted in statistical insignificance (Fig. 3C). Most importantly, the downregulation of *BCL-xL* led to approximately 50% cell death in MDA-MB-231 cells overexpressing miR-222 (Fig. 4D).

### In vivo verification of cell-specific apoptosis in breast cancer xenograft mouse models

Encouraged by the promising results of the miRNA-trigger targeting *BCL-xL* (BCL-xL-222-222), which significantly reduced cell viability in MDA-MB-231 cells, we applied the miRNA-trigger to breast cancer xenograft mouse models. These models were established by subcutaneously injecting 5 × 10^6^ MDA-MB-231 or MDA-MB-453 cells into the backs of athymic nude mice. One week after cell injection, the formation of local tumor nodules was confirmed in all mice. We then administered either the control Luc-222-222 or BCL-xL-222-222 to the xenografted mice via tail-vein injection, using In vivo jetPEI as a delivery vehicle, five times at three-day intervals (Fig. 4A). The therapeutic efficacy of the miRNA-triggers was evaluated by measuring tumor size with a caliper three days after each injection (Fig. 4B). As a result, BCL-xL-222-222 significantly reduced tumor volume in MDA-MB-231 xenograft mice, particularly after the second injection. In contrast, BCL-xL-222-222 showed no therapeutic effect in MDA-MB-453 xenograft mice, whose tumor sizes continued to increase, similar to those injected with the control trigger (Luc-222-222). We observed that the body weights of the xenografted mice were minimally affected by the miRNA-triggers, suggesting that the treatment did not cause significant side effects (Fig. 4C). Three days after the final injection (day 22), the mice were sacrificed, and their tumors were excised for analysis. As shown in Fig. 4D, the tumor sizes in the MDA-MB-231 xenograft mice treated with BCL-xL-222-222 were significantly smaller compared to the other groups. Additionally, miR-222 was highly expressed in the MDA-MB-231 xenograft mice but not in the MDA-MB-453 xenograft mice (Fig. 4E). Furthermore, BCL-xL-222-222 treatment significantly reduced *BCL-xL* mRNA expression levels in the MDA-MB-231 xenografts, whereas no statistically significant change was observed in the MDA-MB-453 xenografts (Fig. 4F). These findings are consistent with the in vitro results and strongly demonstrate that the developed miRNA-trigger can induce cell-specific apoptosis in xenograft mice in a miRNA-dependent manner.

## Conclusions

In this study, we designed and developed a miRNA-trigger to achieve cell-specific gene regulation by leveraging active miRNAs. By incorporating both miRNA and mRNA binding sites, the miRNA-trigger was engineered to hijack disease-specific miRNAs and redirect them to target newly assigned disease-causing genes. We demonstrated the therapeutic potential of this technology by hijacking cancer-specific miRNAs and directing them to the anti-apoptotic *BCL-xL* mRNA, inducing apoptosis in a cell-specific manner. Additionally, we confirmed that our miRNA-trigger functions in vivo, inducing apoptosis specifically in MDA-MB-231 breast cancer cells, which overexpress the breast cancer-specific miR-222, and reducing tumor volume by up to 50% in breast cancer xenograft mouse models.

Most previous RNAi-based gene regulation strategies, which rely on siRNAs or shRNAs [20–22], have limited clinical applications due to unexpected off-target effects. One cause is the short seed sequence within small RNAs, leading to the downregulation of even partially matched mRNAs. Our miRNA-trigger technology addresses this issue by designing the mRNA complementary sequence to be at least 14 nucleotides long, reducing potential off-target effects compared to conventional small RNAs. Additionally, a key advancement of the miRNA-trigger is its exceptional specificity for mediating miRNA. It can distinguish between closely related miRNAs, such as miR-141 and miR-200c, which differ by just one base in their seed sequences. Due to these features, the miRNA-trigger offers a significant improvement over current RNAi technology by exhibiting high specificity in hijacking the intended miRNA and regulating newly assigned target mRNAs.

Our current study focuses on the design and application of the miRNA-trigger for gene regulation to induce cell-specific apoptosis. However, this technology is not limited to treating human diseases and could be extended to address other important biological processes where miRNA profiles play a key role. For instance, during epithelial-mesenchymal transition (EMT), miRNAs such as miR-24, miR-29a, and miR-27a are upregulated to facilitate the process [33–36]. By applying our miRNA-trigger to redirect these mesenchymal miRNAs to target key EMT-promoting genes like *ZEB*, *TWIST*, and *SNAIL*, we may be able to block or significantly delay EMT. Similarly, the miRNA-trigger technology could be used to prevent tumorigenesis by redirecting oncomiRs to target oncogenes. This approach would have minimal impact on normal cells with low oncomiR expression but would become activated in cells undergoing tumorigenesis. Once activated, the triggers would repress oncogenes, effectively preventing tumor formation. Moreover, the miRNA-triggers would stop functioning when oncomiR expression returns to normal, enabling self-regulating therapy. Overall, this work paves the way for the next generation of RNA therapeutics, potentially evolving into novel technologies that can control signal transduction, regulate translation, and even manipulate cell fate with minimal side effects.

## Methods/experimental

### Stability analysis of the miRNA-trigger

Oligonucleotides were incubated in human serum, cell lysates, or distilled water for 24 h at 37 °C. The final concentrations of human serum and cell lysates were 80% and 5 mg ml^−1^, respectively. After incubation, the samples were loaded into a centrifugal filter device (MWCO = 30 kDa, Millipore) and centrifuged at 14,000xg for 30 min. The flow-through, containing oligonucleotides (~ 15 kDa), was collected and resolved on a 15% polyacrylamide gel using 1 × TBE buffer at a constant voltage of 120 V for 120 min. The gel was stained with EtBr and imaged using a UV transilluminator. The sequences of the miRNA-trigger used in this study are listed in Supplementary Table 1.

### Cell culture and transfection

Cell lines were obtained from the American Type Culture Collection (ATCC) or the Korean Cell Line Bank (KCLB). HeLa-141 and HeLa-200c cell lines were gifts from Prof. V. Narry. Kim at Seoul National University. Culture media (Welgene) were supplemented with 10% fetal bovine serum (FBS) (Gibco), and cells were maintained at 37 °C in 5% CO_2_. Cells were routinely tested for mycoplasma contamination, and the authenticity of the cell lines was confirmed via short tandem repeat (STR) profiling. For HeLa-141 and HeLa-200c cells, hygromycin and G418 were added to the media for selection. Transfection was performed using Lipofectamine 3000 (Thermo Fisher Scientific) according to the manufacturer’s instructions. Cells were transfected with siRNA (60nм) or miRNA-trigger (120nм) and incubated for 48–72 h. The sequences of siRNAs used in this study are provided in Supplementary Table 2.

### Western blotting

To prepare total cell lysates, cells were collected using a scraper and lysed in RIPA lysis buffer (150 mм NaCl, 5 mм EDTA, 50 mм Tris, 1% NP-40, 0.5% sodium deoxycholate, 0.1% SDS). A total of 30–40 µg of protein was loaded onto a 12% SDS-PAGE gel and transferred to a PVDF membrane using a semidry transfer system. Western blotting was performed using primary antibodies against Bcl-xL (#2764S, Cell Signaling Technology), GAPDH (#SC-32233, Santa Cruz Biotechnology), and β-Tubulin (TUBB) (#2148, Cell Signaling Technology).

### Quantitative real-time PCR

Total RNA was extracted using TRIzol (Thermo Fisher Scientific) following the manufacturer’s protocol. The extracted nucleic acid was treated with DNase I (TaKaRa), and the purified RNA was reverse transcribed using RevertAid reverse transcriptase (Thermo Fisher Scientific) with random hexamers. The resulting cDNA was subjected to real-time PCR using SYBR Green PCR master mix (Thermo Fisher Scientific) and analyzed on the StepOnePlus real-time PCR system. The primers used in this study are listed in Supplementary Table 3.

For miRNA expression analysis, the universal stem-loop primer method described was employed [37]. This method uses a universal stem-loop primer to elongate the target miRNA during reverse transcription. The universal stem-loop primer sequence is 5′-GAA AGA AGG CGA GGA GCA GAT CGA GGA AGA AGA CGG AAG AAT GTG CGT CTC GCC TTC TTT CNN NNN NNN-3′, where N represents specific sequences of the target miRNA. This stem-loop primer serves as a reverse transcription template for synthesizing cDNA from the target miRNA. The cDNA was then amplified by PCR using SensiFAST SYBR Lo-Rox (Bioline) on the Agilent AriaMx real-time PCR system (Agilent) or the CFX96™ Real-Time System (Bio-Rad). The primers used for miRNA analysis are listed in Supplementary Table 4.

### Cell counting Kit-8 (CCK-8) assay

Cell viability was assessed using the CCK-8 assay (Dojindo) following the manufacturer’s instructions. Cells were seeded in a 96-well plate (5 × 10^3^ cells well^−1^) and cultured for 24 h before being transfected with miRNA-triggers. After three days of incubation, the supernatant was removed, and the cells were incubated in fresh culture media containing CCK-8 solution for 2 h at 37 °C. The optical density at 450 nm was then measured using an Infinite M200 Pro microplate reader (Tecan).

### Animal study

Immune-deficient female athymic nude mice (homozygous Nu-/-) were subcutaneously injected with 5 × 10^6^ breast cancer cells (either miR-222-overexpressing MDA-MB-231 (n = 10) or miR-222-non-overexpressing MDA-MB-453 (n = 10)) suspended in 100 μL of a 1:1 mixture of Matrigel (Corning, NY, USA) and phosphate-buffered saline. On the seventh day after tumor inoculation, tumor formation was confirmed, and the mice were divided into four groups: MDA-MB-231-engrafted mice treated with either the miRNA-trigger BCL-xL-222-222 (n = 5) or Luc-222-222 (n = 5), and MDA-MB-453-engrafted mice treated with BCL-xL-222-222 (n = 5) or Luc-222-222 (n = 5). A dose of 1 mg kg^−1^ of the miRNA-triggers mixed with in vivo-jetPEI (Polyplus-transfection) was injected via tail vein starting on day 7, followed by additional injections every 72 h for a total of five injections. Tumor volume (length × width^3^ / 2) and body weight were measured before each injection. On day 22, the mice were sacrificed, and tumors were excised. The expression levels of miR-222-5p, miR-222-3p, and *BCL-xL* mRNA were measured by RT-qPCR and compared among the groups. *BCL-xL* expression was normalized to *GAPDH* mRNA, and miR-222 expression was normalized to *U6* RNA. The experimental protocol was reviewed and approved by the Institutional Review Board of Seoul National University Hospital, Seoul, Republic of Korea (IRB No. H-1901-073-1003). tuned to the expression profiles of disease-specific miRNA.

## Supplementary Information

Supplementary file 1.

## Data Availability

The datasets used and analyzed during the current study are available from the corresponding author on reasonable request.

## Abbreviations

BCL-xL: B-cell lymphoma-extra-large
ATCC: American Type Culture Collection
KCLB: Korean Cell Line Bank
FBS: Fetal bovine serum
STR: Short tandem repeat
TUBB: β-Tubulin
CCK-8: Cell Counting Kit-8
AGO: Argonaut
PS: Phosphorothioate
2′O-Me: 2′O-methyl
EMT: Epithelial-mesenchymal transition

## References

[1] T. Fehlmann, B. Lehallier, N. Schaum, O. Hahn, M. Kahraman, Y. Li, N. Grammes, L. Geffers, C. Backes, R. Balling, F. Kern, R. Kruger, F. Lammert, N. Ludwig, B. Meder, B. Fromm, W. Maetzler, D. Berg, K. Brockmann, C. Deuschle, A.K. von Thaler, G.W. Eschweiler, S. Milman, N. Barziliai, M. Reichert, T. Wyss-Coray, E. Meese, A. Keller, Common diseases alter the physiological age-related blood microRNA profile. Nat. Commun.Commun. **11**(1), 5958 (2020). 10.1038/s41467-020-19665-1 10.1038/s41467-020-19665-1 PMC7686493 33235214

[2] R.M. O’Connell, D.S. Rao, A.A. Chaudhuri, M.P. Boldin, K.D. Taganov, J. Nicoll, R.L. Paquette, D. Baltimore, Sustained expression of microRNA-155 in hematopoietic stem cells causes a myeloproliferative disorder. J. Exp. Med. **205**(3), 585–594 (2008). 10.1084/jem.20072108 18299402 10.1084/jem.20072108 PMC2275382

[3] R.M. O’Connell, D. Kahn, W.S. Gibson, J.L. Round, R.L. Scholz, A.A. Chaudhuri, M.E. Kahn, D.S. Rao, D. Baltimore, MicroRNA-155 promotes autoimmune inflammation by enhancing inflammatory T cell development. Immunity **33**(4), 607–619 (2010). 10.1016/j.immuni.2010.09.009 20888269 10.1016/j.immuni.2010.09.009 PMC2966521

[4] G.C. van Almen, W. Verhesen, R.E. van Leeuwen, M. van de Vrie, C. Eurlings, M.W. Schellings, M. Swinnen, J.P. Cleutjens, M.A. van Zandvoort, S. Heymans, B. Schroen, MicroRNA-18 and microRNA-19 regulate CTGF and TSP-1 expression in age-related heart failure. Aging Cell **10**(5), 769–779 (2011). 10.1111/j.1474-9726.2011.00714.x 21501375 10.1111/j.1474-9726.2011.00714.x PMC3193380

[5] T. Thum, C. Gross, J. Fiedler, T. Fischer, S. Kissler, M. Bussen, P. Galuppo, S. Just, W. Rottbauer, S. Frantz, M. Castoldi, J. Soutschek, V. Koteliansky, A. Rosenwald, M.A. Basson, J.D. Licht, J.T. Pena, S.H. Rouhanifard, M.U. Muckenthaler, T. Tuschl, G.R. Martin, J. Bauersachs, S. Engelhardt, MicroRNA-21 contributes to myocardial disease by stimulating MAP kinase signalling in fibroblasts. Nature **456**(7224), 980–984 (2008). 10.1038/nature07511 19043405 10.1038/nature07511

[6] A. Care, D. Catalucci, F. Felicetti, D. Bonci, A. Addario, P. Gallo, M.L. Bang, P. Segnalini, Y. Gu, N.D. Dalton, L. Elia, M.V. Latronico, M. Hoydal, C. Autore, M.A. Russo, G.W. Dorn 2nd., O. Ellingsen, P. Ruiz-Lozano, K.L. Peterson, C.M. Croce, C. Peschle, G. Condorelli, MicroRNA-133 controls cardiac hypertrophy. Nat. Med. **13**(5), 613–618 (2007). 10.1038/nm1582 17468766 10.1038/nm1582

[7] X. Li, C. Teng, J. Ma, N. Fu, L. Wang, J. Wen, T.Y. Wang, miR-19 family: a promising biomarker and therapeutic target in heart, vessels and neurons. Life Sci. **232**, 116651 (2019). 10.1016/j.lfs.2019.116651 31302195 10.1016/j.lfs.2019.116651

[8] X. Chen, Y. Ba, L. Ma, X. Cai, Y. Yin, K. Wang, J. Guo, Y. Zhang, J. Chen, X. Guo, Q. Li, X. Li, W. Wang, Y. Zhang, J. Wang, X. Jiang, Y. Xiang, C. Xu, P. Zheng, J. Zhang, R. Li, H. Zhang, X. Shang, T. Gong, G. Ning, J. Wang, K. Zen, J. Zhang, C.Y. Zhang, Characterization of microRNAs in serum: a novel class of biomarkers for diagnosis of cancer and other diseases. Cell Res. **18**(10), 997–1006 (2008). 10.1038/cr.2008.282 18766170 10.1038/cr.2008.282

[9] D.D. Taylor, C. Gercel-Taylor, MicroRNA signatures of tumor-derived exosomes as diagnostic biomarkers of ovarian cancer. Gynecol. Oncol. **110**(1), 13–21 (2008). 10.1016/j.ygyno.2008.04.033 18589210 10.1016/j.ygyno.2008.04.033

[10] M.D. Mattie, C.C. Benz, J. Bowers, K. Sensinger, L. Wong, G.K. Scott, V. Fedele, D. Ginzinger, R. Getts, C. Haqq, Optimized high-throughput microRNA expression profiling provides novel biomarker assessment of clinical prostate and breast cancer biopsies. Mol. Cancer **5**, 24 (2006). 10.1186/1476-4598-5-24 16784538 10.1186/1476-4598-5-24 PMC1563474

[11] R. Duan, Z. Zhang, F. Zheng, L. Wang, J. Guo, T. Zhang, X. Dai, S. Zhang, D. Yang, R. Kuang, G. Wang, C. He, A. Hakeem, C. Shu, P. Yin, X. Lou, F. Zeng, H. Liang, F. Xia, Combining protein and miRNA quantification for bladder cancer analysis. ACS Appl. Mater. Interfaces **9**(28), 23420–23427 (2017). 10.1021/acsami.7b05639 28636312 10.1021/acsami.7b05639

[12] L. Liu, Q. Xu, S. Hao, Y. Chen, A Quasi-direct LC-MS/MS-based targeted proteomics approach for miRNA quantification via a covalently immobilized DNA-peptide probe. Sci. Rep. **7**(1), 5669 (2017). 10.1038/s41598-017-05495-7 28720752 10.1038/s41598-017-05495-7 PMC5515972

[13] P. Androvic, L. Valihrach, J. Elling, R. Sjoback, M. Kubista, Two-tailed RT-qPCR: a novel method for highly accurate miRNA quantification. Nucleic Acids Res. **45**(15), e144 (2017). 10.1093/nar/gkx588 28911110 10.1093/nar/gkx588 PMC5587787

[14] M. El Aamri, G. Yammouri, H. Mohammadi, A. Amine, H. Korri-Youssoufi, Electrochemical biosensors for detection of MicroRNA as a cancer biomarker: pros and cons. Biosensors (Basel). (2020). 10.3390/bios10110186 33233700 10.3390/bios10110186 PMC7699780

[15] R. Rupaimoole, F.J. Slack, MicroRNA therapeutics: towards a new era for the management of cancer and other diseases. Nat. Rev. Drug Discov.Discov. **16**(3), 203–221 (2017). 10.1038/nrd.2016.246 10.1038/nrd.2016.246 28209991

[16] R.R.A. Kadir, M. Alwjwaj, U. Bayraktutan, MicroRNA: an emerging predictive, diagnostic, prognostic and therapeutic strategy in ischaemic stroke. Cell. Mol. Neurobiol.Neurobiol. (2020). 10.1007/s10571-020-01028-5 10.1007/s10571-020-01028-5 PMC9142420 33368054

[17] A.L. Jackson, S.R. Bartz, J. Schelter, S.V. Kobayashi, J. Burchard, M. Mao, B. Li, G. Cavet, P.S. Linsley, Expression profiling reveals off-target gene regulation by RNAi. Nat. Biotechnol. Biotechnol. **21**(6), 635–637 (2003). 10.1038/nbt831 10.1038/nbt831 12754523

[18] Y. Fedorov, E.M. Anderson, A. Birmingham, A. Reynolds, J. Karpilow, K. Robinson, D. Leake, W.S. Marshall, A. Khvorova, Off-target effects by siRNA can induce toxic phenotype. RNA **12**(7), 1188–1196 (2006). 10.1261/rna.28106 16682561 10.1261/rna.28106 PMC1484448

[19] G. Pepin, J. Ferrand, M.P. Gantier, Assessing the off-target effects of miRNA inhibitors on innate immune toll-like receptors. Methods Mol. Biol. **1517**, 127–135 (2017). 10.1007/978-1-4939-6563-2_9 27924479 10.1007/978-1-4939-6563-2_9

[20] R.W. Carthew, E.J. Sontheimer, Origins and mechanisms of miRNAs and siRNAs. Cell **136**(4), 642–655 (2009). 10.1016/j.cell.2009.01.035 19239886 10.1016/j.cell.2009.01.035 PMC2675692

[21] D.D. Rao, J.S. Vorhies, N. Senzer, J. Nemunaitis, siRNA vs. shRNA: similarities and differences. Adv. Drug Deliv. Rev.Deliv Rev. **61**(9), 746–759 (2009). 10.1016/j.addr.2009.04.004 10.1016/j.addr.2009.04.004 19389436

[22] J.C. Burnett, J.J. Rossi, K. Tiemann, Current progress of siRNA/shRNA therapeutics in clinical trials. Biotechnol. J.. J. **6**(9), 1130–1146 (2011). 10.1002/biot.201100054 10.1002/biot.201100054 PMC3388104 21744502

[23] R.S. Pillai, C.G. Artus, W. Filipowicz, Tethering of human Ago proteins to mRNA mimics the miRNA-mediated repression of protein synthesis. RNA **10**(10), 1518–1525 (2004). 10.1261/rna.7131604 15337849 10.1261/rna.7131604 PMC1370638

[24] Y. Kim, J.H. Lee, J.E. Park, J. Cho, H. Yi, V.N. Kim, PKR is activated by cellular dsRNAs during mitosis and acts as a mitotic regulator. Genes Dev. **28**(12), 1310–1322 (2014). 10.1101/gad.242644.114 24939934 10.1101/gad.242644.114 PMC4066401

[25] L.V. Croft, M. Fisher, T.K. Barbhuiya, S. El-Kamand, S. Beard, A. Rajapakse, R. Gamsjaeger, L. Cubeddu, E. Bolderson, K. O’Byrne, D. Richard, N.S. Gandhi, Sequence- and structure-dependent cytotoxicity of phosphorothioate and 2’-O-methyl modified single-stranded oligonucleotides. Nucleic Acid Ther.Ther. **34**(3), 143–155 (2024). 10.1089/nat.2023.0056 10.1089/nat.2023.0056 38648015

[26] M.M. Janas, Y. Jiang, M.K. Schlegel, S. Waldron, S. Kuchimanchi, S.A. Barros, Impact of oligonucleotide structure, chemistry, and delivery method on in vitro cytotoxicity. Nucleic Acid Ther.Ther. **27**(1), 11–22 (2017). 10.1089/nat.2016.0639 10.1089/nat.2016.0639 27923110

[27] A.J. Souers, J.D. Leverson, E.R. Boghaert, S.L. Ackler, N.D. Catron, J. Chen, B.D. Dayton, H. Ding, S.H. Enschede, W.J. Fairbrother, D.C. Huang, S.G. Hymowitz, S. Jin, S.L. Khaw, P.J. Kovar, L.T. Lam, J. Lee, H.L. Maecker, K.C. Marsh, K.D. Mason, M.J. Mitten, P.M. Nimmer, A. Oleksijew, C.H. Park, C.M. Park, D.C. Phillips, A.W. Roberts, D. Sampath, J.F. Seymour, M.L. Smith, G.M. Sullivan, S.K. Tahir, C. Tse, M.D. Wendt, Y. Xiao, J.C. Xue, H. Zhang, R.A. Humerickhouse, S.H. Rosenberg, S.W. Elmore, ABT-199, a potent and selective BCL-2 inhibitor, achieves antitumor activity while sparing platelets. Nat. Med. **19**(2), 202–208 (2013). 10.1038/nm.3048 23291630 10.1038/nm.3048

[28] M.M. Williams, R.S. Cook, Bcl-2 family proteins in breast development and cancer: could Mcl-1 targeting overcome therapeutic resistance? Oncotarget **6**(6), 3519–3530 (2015). 10.18632/oncotarget.2792 25784482 10.18632/oncotarget.2792 PMC4414133

[29] T. Oltersdorf, S.W. Elmore, A.R. Shoemaker, R.C. Armstrong, D.J. Augeri, B.A. Belli, M. Bruncko, T.L. Deckwerth, J. Dinges, P.J. Hajduk, M.K. Joseph, S. Kitada, S.J. Korsmeyer, A.R. Kunzer, A. Letai, C. Li, M.J. Mitten, D.G. Nettesheim, S. Ng, P.M. Nimmer, J.M. O’Connor, A. Oleksijew, A.M. Petros, J.C. Reed, W. Shen, S.K. Tahir, C.B. Thompson, K.J. Tomaselli, B. Wang, M.D. Wendt, H. Zhang, S.W. Fesik, S.H. Rosenberg, An inhibitor of Bcl-2 family proteins induces regression of solid tumours. Nature **435**(7042), 677–681 (2005). 10.1038/nature03579 15902208 10.1038/nature03579

[30] J. Li, J. Viallet, E.B. Haura, A small molecule pan-Bcl-2 family inhibitor, GX15-070, induces apoptosis and enhances cisplatin-induced apoptosis in non-small cell lung cancer cells. Cancer Chemother. Pharmacol.Pharmacol. **61**(3), 525–534 (2008). 10.1007/s00280-007-0499-3 10.1007/s00280-007-0499-3 17505826

[31] H. Tamaki, N. Harashima, M. Hiraki, N. Arichi, N. Nishimura, H. Shiina, K. Naora, M. Harada, Bcl-2 family inhibition sensitizes human prostate cancer cells to docetaxel and promotes unexpected apoptosis under caspase-9 inhibition. Oncotarget **5**(22), 11399–11412 (2014). 10.18632/oncotarget.2550 25333266 10.18632/oncotarget.2550 PMC4294332

[32] M. Riaz, M.T. van Jaarsveld, A. Hollestelle, W.J. Prager-van der Smissen, A.A. Heine, A.W. Boersma, J. Liu, J. Helmijr, B. Ozturk, M. Smid, E.A. Wiemer, J.A. Foekens, J.W. Martens, miRNA expression profiling of 51 human breast cancer cell lines reveals subtype and driver mutation-specific miRNAs. Breast Cancer Res. **15**(2), R33 (2013). 10.1186/bcr3415 23601657 10.1186/bcr3415 PMC3672661

[33] J. Zhang, L. Ma, MicroRNA control of epithelial-mesenchymal transition and metastasis. Cancer Metastasis Rev. **31**(3–4), 653–662 (2012). 10.1007/s10555-012-9368-6 22684369 10.1007/s10555-012-9368-6 PMC3686549

[34] H. Listing, W.A. Mardin, S. Wohlfromm, S.T. Mees, J. Haier, MiR-23a/-24-induced gene silencing results in mesothelial cell integration of pancreatic cancer. Br. J. Cancer **112**(1), 131–139 (2015). 10.1038/bjc.2014.587 25422915 10.1038/bjc.2014.587 PMC4453619

[35] Y. Wu, W. Shi, T. Tang, Y. Wang, X. Yin, Y. Chen, Y. Zhang, Y. Xing, Y. Shen, T. Xia, C. Guo, Y. Pan, L. Jin, miR-29a contributes to breast cancer cells epithelial-mesenchymal transition, migration, and invasion via down-regulating histone H4K20 trimethylation through directly targeting SUV420H2. Cell Death Dis. **10**(3), 176 (2019). 10.1038/s41419-019-1437-0 30792382 10.1038/s41419-019-1437-0 PMC6385178

[36] G. Jiang, W. Shi, H. Fang, X. Zhang, miR-27a promotes human breast cancer cell migration by inducing EMT in a FBXW7-dependent manner. Mol. Med. Rep. **18**(6), 5417–5426 (2018). 10.3892/mmr.2018.9587 30365154 10.3892/mmr.2018.9587 PMC6236270

[37] L.H. Yang, S.L. Wang, L.L. Tang, B. Liu, W.L. Ye, L.L. Wang, Z.Y. Wang, M.T. Zhou, B.C. Chen, Universal stem-loop primer method for screening and quantification of microRNA. PLoS ONE **9**(12), e115293 (2014). 10.1371/journal.pone.0115293 25548906 10.1371/journal.pone.0115293 PMC4280144

