# Supplementary material for: Self-modulating therapeutic platform using engineered miRNA-responsive oligonucleotides
Source: Nano Converg. 2025 Jun 30;12:32. doi: 10.1186/s40580-025-00499-w (PMC12209101; doi:10.1186/s40580-025-00499-w)
Supplement: Supplementary file 1 [file 40580_2025_499_MOESM1_ESM.pdf]

Supporting Information

Self-Modulating Therapeutic Platform Using Engineered miRNA-Responsive Oligonucleotides

Doyeong Ku[1†], Hansol Kim[1†], JinA Lim[1], Jayeon Song[1], Junhyeok Yoon[1], Liu Jin[2, 3], Su-Ji Min[2], Ryeongeun Cho[1], Namseok Lee[1], Kyunghoon Hur[1], Jong-Eun Park[4], Luke P. Lee[5, 6, 7], Junshik Hong[2, 3, 8], Yoosik Kim[1, 9, 10], and Hyun Gyu Park[1, 9*]

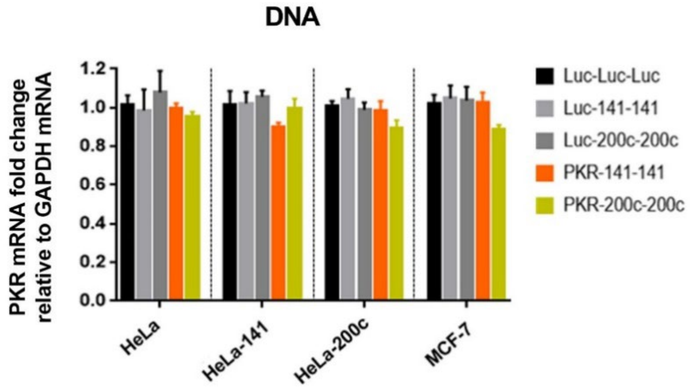

Figure S1. *GAPDH*-normalized *PKR* mRNA expression in HeLa, HeLa-141, HeLa-200c, and MCF-7 cells after transfection with unmodified DNA miRNA-triggers targeting *PKR* mRNA.

Trigger name	Sequence (5’-3’)
**DNA trigger**	TAT TTC TCA TTC CCC CAT CTT TAC CAG ACA GTG TTA GGG AAT GAG AAA TA
**LNA trigger**	[LNA(T)][LNA(A)][LNA(T)] [LNA(T)]TC TCA TTC CCC CAT CTT TAC CAG ACA GTG TTA GGG AAT GAG A[LNA(A)][LNA(A)] [LNA(T)][LNA(A)]
**PS trigger (4)**	T*A*T *T*TC TCA TTC CCC CAT CTT TAC CAG ACA GTG TTA GGG AAT GAG A*A*A*T*A
**PS trigger (8)**	T*A*T *T*T*C *T*C*A TTC CCC CAT CTT TAC CAG ACA GTG TTA GGG AAT *G*A*G *A*A*A *T*A
**FPS trigger**	T*A*T* T*T*C* T*C*A* T*T*C* C*C*C* C*A*T* C*T*T* T*A*C* C*A*G* A*C*A* G*T*G* T*T*A* G*G*G* A*A*T* G*A*G* A*A*A* T*A
**2'O-Me trigger**	mUmAmU mUmUmC mUmCmA mUmUmC mCmCmC mCmAmU mCmUmU mUmAmC mCmAmG mAmCmA mGmUmG mUmUmA mGmGmG mAmAmU mGmAmG mAmAmA mUmA
**Luc-141-141 (DNA)**	CCA TCT TTA CCT CAC AGT GTT A CCA TCT TTA CCT CAC AGT GTT A TCG AAG TAC TCA GCG TAA G
**Luc-Luc-Luc (DNA)**	TCG AAG TAC TCA GCG TAA GTC GAA GTA CTC AGC GTA AGT CGA AGT ACT CAG CGT AAG
**Luc-200c-200c (DNA)**	TCC ATC ATT ACC GCG CAG TAT TAT CCA TCA TTA CCG CGC AGT ATT ATC GAA GTA CTC AGC GTA AG
**PKR-141-141 (DNA)**	CCA TCT TTA CCT CAC AGT GTT ACC ATC TTT ACC TCA CAG TGT TAT ATG TGA GGC AGA GAA CGA
**PKR-200c-200c (DNA)**	TCC ATC ATT ACC GCG CAG TAT TA TCC ATC ATT ACC GCG CAG TAT TA TAT GTG AGG CAG AGA ACG A
**Luc-Luc-Luc (FPS)**	T*C*G *A*A*G *T*A*C *T*C*A *G*C*G *T*A*A *G*T*C *G*A*A *G*T*A *C*T*C *A*G*C *G*T*A *A*G*T *C*G*A *A*G*T *A*C*T *C*A*G *C*G*T *A*A*G
**Luc-141-141 (FPS)**	*C*C*A *T*C*T *T*T*A *C*C*T *C*A*C *A*G*T *G*T*T *A*C*C *A*T*C *T*T*T *A*C*C *T*C*A *C*A*G *T*G*T *T*A*T *T*A*T *G*T*T *T*C*A *G*G*T *T*C*A *G*G*G *G*G*A
**Luc-200c-200c (FPS)**	*T*C*C *A*T*C *A*T*T *A*C*C *G*C*G *C*A*G *T*A*T *T*A*T *C*C*A *T*C*A *T*T*A *C*C*G *C*G*C *A*G*T *A*T*T *A*T*C *G*A*A *G*T*A *C*T*C *A*G*C *G*T*A *A*G
**PKR-141-141 (FPS)**	*C*C*A *T*C*T *T*T*A *C*C*T *C*A*C *A*G*T *G*T*T *A*C*C *A*T*C *T*T*T *A*C*C *T*C*A *C*A*G *T*G*T *T*A*T *A*T*G *T*G*A *G*G*C *A*G*A *G*A*A *C*G*A
**PKR-200c-200c (FPS)**	*T*C*C *A*T*C *A*T*T *A*C*C *G*C*G *C*A*G *T*A*T *T*A *T*C*C *A*T*C *A*T*T *A*C*C *G*C*G *C*A*G *T*A*T *T*A *T*A*T *G*T*G *A*G*G *C*A*G *A*G*A *A*C*G *A
**Luc-Luc-Luc (2’O-Me)**	mUmCmG mAmAmG mUmAmC mUmCmA mGmCmG mUmAmA mGmUmC mGmAmA mGmUmA mCmUmC mAmGmC mGmUmA mAmGmU mCmGmA mAmGmU mAmCmU mCmAmG mCmGmU mAmAmG
**Luc-141-141 (2’O-Me)**	mCmCmA mUmCmU mUmUmA mCmCmU mCmAmC mAmGmU mGmUmU mAmCmC mAmUmC mUmUmU mAmCmC mUmCmA mCmAmG mUmGmU mUmAmU mCmGmA mAmGmU mAmCmU mCmAmG mCmGmU mAmAmG
**Luc-200c-200c (2’O-Me)**	mUmCmC mAmUmC mAmUmU mAmCmC mGmCmG mCmAmG mUmAmU mUmAmU mCmCmA mUmCmA mUmUmA mCmCmG mCmGmC mAmGmU mAmUmU mAmUmC mGmAmA mGmUmA mCmUmC mAmGmC mGmUmA mAmG
**PKR-141-141 (2’O-Me)**	mCmCmA mUmCmU mUmUmA mCmCmU mCmAmC mAmGmU mGmUmU mAmCmC mAmUmC mUmUmU mAmCmC mUmCmA mCmAmG mUmGmU mUmAmU mAmUmG mUmGmA mGmGmC mAmGmA mGmAmA mCmGmA

**PKR-200c-200c (2’O-Me)**	mUmCmC mAmUmC mAmUmU mAmCmC mGmCmG mCmAmG mUmAmU mUmA mUmCmC mAmUmC mAmUmU mAmCmC mGmCmG mCmAmG mUmAmU mUmA mUmAmU mGmUmG mAmGmG mCmAmG mAmGmA mAmCmG mA
**Luc-222-222**	mAmCmC mCmAmG mUmAmG mCmCmA mGmAmU mGmUmA mGmCmU mAmCmC mCmAmG mUmAmG mCmCmA mGmAmU mGmUmA mGmCmU mUmCmG mAmAmG mUmAmC mUmCmA mGmCmG mUmAmA mG
**BCL-xL-222-222**	mAmCmC mCmAmG mUmAmG mCmCmA mGmAmU mGmUmA mGmCmU mAmCmC mCmAmG mUmAmG mCmCmA mGmAmU mGmUmA mGmCmU mUmCmU mCmCmU mUmCmC mUmGmC mCmCmU mUmCmC mU
[LNA(N)] represents locked nucleic acid (LNA) modification; ‘*’ represents phosphorothioate modification; ‘mN’ represents 2'-O-ethyl modification.	

Supp1ementary Table 1. Sequences of the miRNA-triggers.

Gene	Sense(5'-3')	Antisense (5'-3')
siLuc	CUU ACG CUG AGU ACU UCG A	UCG AAG UAC UCA GCG UAA G
siPKR-1	GCA GGG AGU AGU ACU UAA A	UUU AAG UAC UAC UCC CUG C
siPKR-2	GCA UGG GCC AGA AGG AUU U	AAA UCC UUC UGG CCC AUG C
siPKR-3	GCA GAU ACA UCA GAG AUA A	UUA UCU CUG AUG UAU CUG C
siPKR-4	CCU GAG ACC AGU GAU GAU U	AAU CAU CAC UGG UCU CAG G

Supp1ementary Table 2. siRNA sequences.

Target name	Primer name	Sequence (5’-3’)
**GAPDH**	GAPDH-Forward	CTC CTC CAC CTT TGA CGC TG
	GAPDH-Reverse	TCC TCT TGT GCT CTT GCT GG
**PKR**	PKR-Forward	GAG GGG AAT GAT GTG ATT GG
	PKR-Reverse	CTG GGC TGT CAC TTC TAG CC
**BCL-xL**	BCL-xL-Forward	TCC CCA TGG CAG CAG TAA AG
	BCL-xL-Reverse	TCC ACA AAA GTA TCC TGT TCA AAG C

Supp1ementary Table 3. Primer sequences of RT-qPCR.

Primer name	Sequence (5’-3’)
**U6_RT Primer**	CGC TTC ACG AAT TTG CGT GTC AT
**U6_Forward Primer**	GCT TCG GCA GCA CAT ATA CTA AAA T
**U6_Reverse Primer**	CGC TTC ACG AAT TTG CGT GTC AT
**miR-141_RT primer**	CTC AAC TGG TGT CGT GGA GTC GGC AAT TCA GTT GAG CCA TCT TT
**miR-141_Forward Primer**	ACA CTC CAG CTG GGT AAC ACT GTC TGG TAA
**miR-141_Reverse Primer**	CTC AAC TGG TGT CGT GGA GTC GGC AA
**miR-200c_RT Primer**	CTC GTA TCC AGT GCA GGG TCC GAG GTA TTC GCA CTG GAT ACG AGC CAA AC
**miR-200c_Forward Primer**	GAG CCG TCT TAC CCA GCA
**miR-200c_Reverse Primer**	GTG CAG GGT CCG AGG TAT
**miR-222_RT Primer**	GAA AGA AGG CGA GGA GCA GAT CGA GGA AGA AGA CGG AAG AAT GTG CGT CTC GCC TTC TTT CAC CCA GTA
**miR-222_Forward Primer**	AGC TAC ATC TGG CTA CTG GGT
**miR-222_Reverse Primer**	CGA GGA AGA AGA CGG AAG AAT
**miR-21 _RT Primer**	GTC GTA TCC AGT GCA GGG TCC GAG GTA TTC GCA CTG GAT ACG ACT CAA CA
**miR-21 _Forward Primer**	GCC CGC TAG CTT ATC AGA CTG ATG
**miT-21 _Reverse Primer**	GTG CAG GGT CCG AGG T

Supp1ementary Table 4. Primer sequences for the stem-loop RT-qPCR.
